# A systematic review and meta-analysis of the diagnostic accuracy of the Phototest for cognitive impairment and dementia

**DOI:** 10.1590/S1980-57642014DN82000009

**Published:** 2014

**Authors:** Cristóbal Carnero-Pardo, Samuel Lopez-Alcalde, Ricardo Francisco Allegri, María Julieta Russo

**Affiliations:** 1 Cognitive Behavioral Neurology Unit, Service of Neurology, Hospital Universitario Virgen de las Nieves, Granada, Spain.; 2 FIDYAN Neurocenter, Granada, España.; 3 Department of Cognitive Neurology, Instituto de Investigaciones Neurológicas Raúl Carrea (FLENI), Buenos Aires, Argentina.

**Keywords:** meta-analysis, screening, detection, cognitive impairment, phototest, dementia

## Abstract

The recently developed Phototest is a simple, easy and very brief test for detecting cognitive impairment or dementia.

**Objective:**

To evaluate the diagnostic accuracy of the Phototest for detecting cognitive impairment or dementia.

**Methods:**

We used a manually created database to search for studies evaluating the Phototest diagnostic yield and performed an initial meta-analysis to determine sensitivity (Sn) and specificity (Sp) of diagnostic parameters. We also performed a second meta-analysis of individual participant data.

**Results:**

In total, 6 studies were included in the meta-analysis. For dementia, Sn was 0.85 (95% CI, 0.82-0.88) and Sp 0.87 (95% CI, 0.85-0.99); for cognitive impairment, Sn was 0.80 (95% CI, 0.77-0.92) and Sp 0.88 (95% CI, 0.86-0.90). In the individual data meta-analysis, 1565 subjects were included, where best cut-off points for dementia and for cognitive impairment were 26/27 (Sn=0.89 (95% CI 0.85-0.91), Sp=0.84 (95% CI, 0.82-0.91)) and 28/29 (Sn=0.79 (95% CI, 0.76-0.81), Sp=0.88 (95% CI, 0.86-0.90)), respectively.

**Conclusion:**

Phototest has good diagnostic accuracy for dementia and cognitive impairment. It is brief, simple and can be used in illiterate persons. This makes it suitable for use in primary care settings and/or in subjects with low educational level.

## Abstract (pt)

Phototest é um teste simples, fácil e muito rápido para detecção de comprometimento cognitivo e demência recentemente desenvolvido.

**Objetivo:**

Avaliar a acurácia diagnostica do Phototest para detecção de comprometimento cognitivo e demência.

**Métodos:**

Nós usamos um banco de dados manualmente criado para estudos que avaliassem a capacidade diagnóstica do Phototest e realizamos uma meta-análise para determinar a sensibilidade (Sn) e especificidade (Ep) dos parâmetros diagnósticos. Nós também realizamos uma segunda meta-análise dos dados individuais dos participantes.

**Resultados:**

Um total de seis estudos foram incluídos na meta-análise. Para demência a Sn foi 0.85 (95% CI, 0,82-0,88) e Ep 0,87 (95% CI, 0,85-0,99); para comprometimento cognitivo a Sn foi 0,80 (95% CI, 0,77-0,92) e Sp 0,88 (95% CI, 0,-0,90). Na meta-análise de dados individuais, 1565 foram incluídos, os melhores escores de corte para demência e para comprometimento cognitivo foram 26/27 (Sn=0,89 (95% CI 0,85-0,91), Ep=0,84 (95% CI, 0,82-0,91)) e 28/29 (Sn=0,79 (95% CI, 0,76-0,81), Ep=0,88 (95% CI, 0,86-0,90)), respectivamente.

**Conclusão:**

Photest tem boa acurácia diagnostica para demência e comprometimento cognitivo. É breve, simples e pode ser usado em pessoas analfabetas. Tornando-o apropriado para o uso em cuidados primários e/ou sujeitos com baixo nível educacional.

## INTRODUCTION

The Phototest (http://www.phototest.es) is a recently developed cognitive test with theoretical advantages over other available dementia screening tests: it is simple and very brief (<3 minutes), can be applied to illiterates, and has results that are not influenced by the subject's educational level.^1^

The test comprises three parts.^2^ First, a naming task with a sheet including the six color photographs of common objects in prototypic position corresponding to different categories (games, vehicles, musical instruments, clothes and eating utensils) is administered. These pictured objects vary in frequency, from high (car) to low frequency (trumpet); in semantic set size, from broad (fruit) to narrow (cutlery); as well as in whether they are prototypical (spoon) or atypical (shoes) elements in a given semantic field. The sheet is then removed. The second task is a verbal fluency test in which participants are asked to say as many opposite-gender names as possible in thirty seconds, and then same-gender ones during the same time period. Unlike other frequently used verbal fluency tests (e.g. animal verbal fluency), this task was shown to be uninfluenced by educational level.^3^ Following the verbal fluency test (names of people), which also has a significant distractor effect between naming and recall tasks; subjects are asked to freely recall the six photographs in any order. After 20 seconds, the category cues are presented to elicit cued recall of only those items that are not retrieved by free recall. In summary, the Phototest assesses multiple cognitive domains including language (naming objects), executive functions (verbal fluency) as well as episodic memory (free recall and cued recall), which show high sensitivity for detecting cognitive impairment (CI) in general, and Alzheimer disease (AD) in particular.^4^

Several studies have assessed the diagnostic accuracy of the Phototest for detecting cognitive impairment and dementia in different settings and sites; however, to date, no meta-analysis of these studies has been performed. The objective of this systematic review and meta-analysis was to evaluate the diagnostic accuracy of the Phototest for detecting cognitive impairment and/or dementia.

## METHODS

**Search criteria for systematic review.** A database of published and unpublished literature was assembled from systematic searches of electronic sources, hand searching, and consultation with experts in the field. The following databases were searched: MEDLINE, PsycINFO, EmBASE, SCIELO, and IBECS. In addition, information on studies in progress, unpublished research or research reported in the grey literature was sought by searching a range of relevant databases including Inside Conferences and Dissertation Abstracts. All studies published from 1 January 2004 through 31 December 2013 that evaluated the diagnostic accuracy of the Phototest for detecting cognitive impairment or dementia, were identified. The search was restricted to English and Spanish language literature. With use of a Boolean strategy, cross searching of the following four categories was done: 1- test ("Fototest" OR "Test de las Fotos" OR "Phototest" OR "Photo-Test"), 2- disease ("cognitive impairment" OR "dementia" or "Alzheimer´s disease"), 3- estimates of diagnostic test accuracy ("sensitivity" OR "specificity" OR "accuracy"), and search terms representing screening tests ("screening" OR "detection"). Furthermore, additional articles were identified by manually searching the authors' own literature databases.

**Inclusion criteria and selection process.** To be included in the review, studies had to meet the following criteria:

[1] population: cognitively healthy older adults or adults with cognitive impairment or dementia according to validated reference standard diagnostic criteria;
[2] outcome: diagnostic accuracy of the Phototest; and
[3] reported data: sensitivity (Sn) and specificity (Sp) values; when unavailable, raw data from the articles were used to construct 2x2 tables. Authors of individual reports were contacted to verify data extracted from the original database and to provide supplementary information pertaining to the criteria used for diagnosing cognitive impairment or dementia.

We developed a data extraction sheet. One review author extracted, or calculated from each study, data on the sensitivity and specificity of the Phototest while a second author checked the extracted data.

**Meta-analysis.** For included studies, our primary outcomes of interest were Sn and Sp at a given cut-off for the Phototest. We did not focus on positive and negative predictive values because the prevalence of cognitive impairment varied widely across studies. We synthesized results for test performance to detect:

[1] dementia; and
[2] cognitive impairment (MCI and/or dementia).

We ran a random effect meta-analysis (Dersimonian-Laird method) for sensitivity and specificity for both groups, using the Meta-Disc^11,5^ program. Heterogeneity was assessed by the Cochran Q test. We also performed a meta-analysis of participants including data from authors' own databases, for which we estimated the area under the ROC curve (aROC), Youden index, Sn and Sp values, and positive and negative likelihood ratios (+LR, -LR), and 95% confidence intervals were calculated for all studied variables. Finally, specific LRs were calculated for different score intervals.

The PRISMA-statement was followed for reporting items of this systematic review and meta-analyses.^6^

## RESULTS

**Figure 1:**
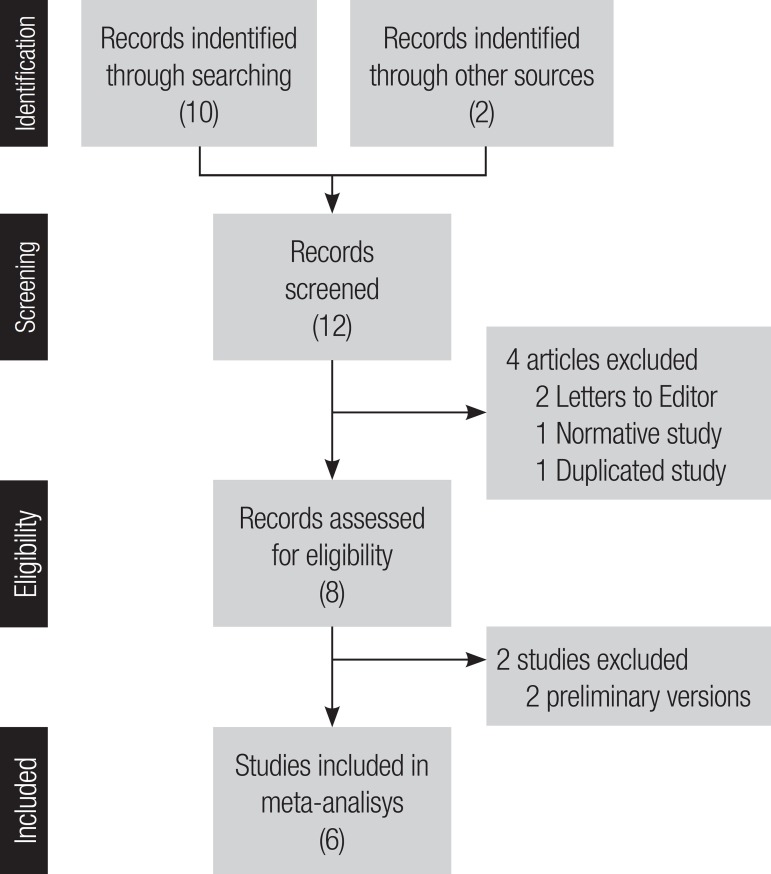
Study selection flow chart based on PRISMA-statement.

**Literature search.** The literature search yielded a total of 10 potentially relevant articles. We also included 2 additional studies conducted in Argentina (an unpublished relevant study^7^ and another ongoing study)^8^ (Figure 1). In total, the articles identified were: two letters to the editor,^9,10^ one normative study^11^ and nine diagnostic test accuracy studies.^1,2,7,8,12-16^ Eight references met the inclusion criteria for this systematic review. Four studies were excluded for the following reasons: letter to editor (n=2),^9,10^ duplicated study (n=1),^14^ and normative data study (n=1).^11^ Finally, two studies were excluded from the meta-analysis since they involved a preliminary version of the Phototest (n=2).^1,7^

**Diagnostic criteria.** All studies from Spain met the Diagnostic and Statistical Manual of Mental Disorders-Fourth Edition, Text Revision (DSM-IV-TR)^17^ criteria for dementia diagnosis, and the recommendations from the Spanish Neurological Society for mild cognitive impairment diagnosis (MCI)^18^. In the Argentine study, only subjects who met the National Institute of Neurological and Communicative Disorders and Stroke-Alzheimer's Disease and Related Disorders Association (NINDS-ADRDA)^19^ criteria for probable Alzheimer-type dementia, the DSM-IV-TR^17^ criteria for dementia, and the conventional Petersen criteria^20^ for single domain amnestic MCI were included.

**Characteristics of included studies.** The systematic review included 8 articles (n=1939 participants, ranging from 60 to 589 subjects). The details of the included studies are summarized in Table 1 and explained below:

**Table 1:** Description of individual studies of diagnostic accuracy of the Phototest included in the meta-analysis

Study, year	Site	Setting	n	Dementia				Cognitive Impairment				Meta-analyses	
				Prevalence	Sn	Sp		Prevalence	Sn	Sp		Aggregate data	Individual data
Carnero C, Montoro MT, 2004	Granada/Barcelona	Occupational care Residential care	60	0.50	0.93	0.80		-	-	-		No	No
Carnero-Pardo C, et al., 2007	Granada	Specialized care	378	0.25	0.88	0.90		0.49	0.90	0.90		Yes	Yes
Barreto MD, et al., 2007	Buenos Aires	Residential care	73	0.16	0.85	0.96		0.37	0.83	0.90		No	No
Baos Sánchez L, et al., 2008	Ciudad Real	Community	261	0.10	0.84	0.85		-	-	-		Yes	No
Carnero-Pardo C, et al., 2011	Granada	Primary careSpecialized care	138	0.34	0.81	0.89		0.60	0.71	0.84		Yes	Yes
Carnero-Pardo C, et al., 2012	Multicenter	Neurological care	589	0.20	0.88	0.87		0.39	0.69	0.93		Yes	Yes
Carnero-Pardo C, et al., 2013	Granada	Specialized care	313	0.45	0.84	0.90		0.70	0.83	0.96		Yes	Yes
Russo MJ, et al., 2012	Buenos Aires	Specialized care	127	0.44	0.79	0.73		0.80	0.90	0.90		Yes	Yes

Sn: sensitivity; Sp: specificity.

*Preliminary Study^1^* - Case-control study providing "proof of concept" of the Phototest. This early version of the test used a sheet with 6 photographs of objects, different to those in the current version, therefore the study was excluded from the current analysis.

*Cross-sectional Study^2^* - Cross-sectional prospective study of subjects attending a Cognitive-Behavioral Neurological Unit. The reference standard (clinical diagnosis) was independent and blind to the Phototest results.

*Argentine-1 Study^7^* - Cross-sectional study performed in a subgroup of patients with established diagnosis, attending elderly day care centers or nursing homes. This study was excluded because it used the earlier version of the Phototest.

*Ciudad Real Study^12^* - Cross-sectional study performed in a simple random sample of individuals corresponding to 10 primary care consultations at Health Center I in Ciudad Real. Only presence or absence of dementia was considered. Diagnosis and the Phototest application were carried out by independent investigators. Individual results for this study were not available.

*FOTOTRANS Study^15^* - Cross-sectional multi-center naturalistic study conducted in individuals with previous diagnosis during 19 visits to General Neurology Departments.

*Granada Study^13^* - Prospective study of consecutive patients suspected of dementia attending four health centers in Granada. The reference standard (clinical diagnosis) was established by blinded trained neurologists from Cognitive-Behavioral Neurology Unit.

*AD^8^ Study^16^* - Cross-sectional, prospective study conducted at a Cognitive-Behavioral Neurology Unit, which served as validation of the Spanish version of the AD^8^ questionnaire (20). In this study, the Phototest was used as a short cognitive reference test to compare and assess construct validity of the AD^8^ questionnaire.^21^

*Argentine-2 Study^8^* - Cross-sectional study of elderly clinical patients attending two memory clinics in Argentina, selected by convenience sampling of consecutive patients suspected of cognitive impairment or dementia.

**Cut-off points.** The optimal cut-off scores for identifying dementia in the studies included varied between 24/25 and 26/27 (positive/ negative). For discriminating between control and cognitive impairment subjects, the optimal cut-off score of the Phototest was 28/29 in the Spanish study and 30/31 in the Argentine sample.

Apart from published information, all individual data were available for 5 out of the 6 included studies^2,8,13,15,16^ and a meta-analysis of individual participant data was performed.

### Meta-analysis

**Figure 2:**
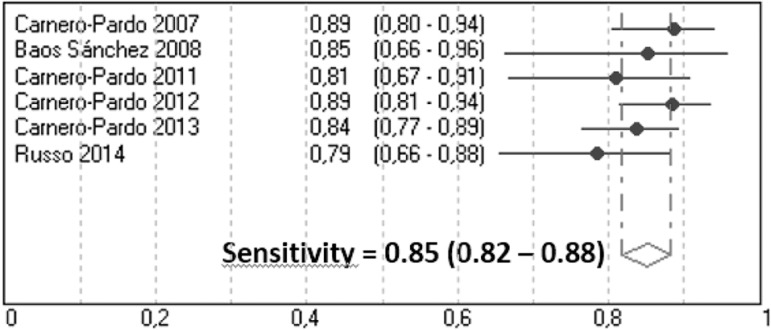
Meta-analysis of sensitivity for dementia.

**Figure 3:**
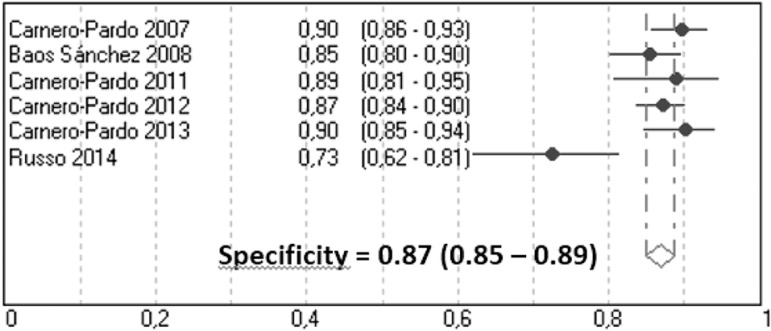
Meta-analysis of specificity for dementia.

*Dementia -* The overall prevalence of dementia for studies in different settings was 30.5 %. Sn and Sp diagnostic parameters for dementia are shown in Table 1. For all dementia subjects, the pooled estimate of Sn was 0.85 (95% CI, 0.82-0.88) with no evidence of significant heterogeneity between studies (Q=4.71, p=0.32) (Figure 2); and the pooled estimate of Sp was 0.87 (95% CI, 0.85-0.89) with evidence of significant heterogeneity (Q=17.3, p<0.01) (Figure 3).

In the meta-analysis of individual participant data, 1565 subjects were included (1104 without dementia and 461 with dementia); the aROC was 0.94 (95% CI, 0.93-0.95) and the value that maximizes the sum of Sn and Sp was 26/27, with Sn and Sp of 0.89 (95% CI, 0.85-0.91) and 0.84 (95% CI, 0.82-0.86), respectively. Diagnostic accuracy parameters (Sn, Sp, +LR, -LR and Youden index) for different cut-offs of pooled individual data are shown in Table 2.

**Table 2:** Results of the meta-analysis of individual participant data based on parameters estimated for dementia.

Cut-off	Sn (95 %CI )	Sp (95 %CI )	+LR (95 %CI )	-LR (95 %CI )	Youden index
23/24	0.71(0.67-0.75)	0.94 (0.92-0.95)	11.72 (9.2-14.9)	0.31 (0.3-0.4)	0.65
24/25	0.78 (0.74-0.82)	0.92 (0.90-0.94)	10.00 (8.1-12.3)	0.24 (0.2-0.3)	0.70
25/26	0.82 (0.79-0.86)	0.88 (0.82-0.90)	7.11 (6.0-8.4)	0.20 (0.2-0.2)	0.70
26/27	0.89 (0.86-0.92)	0.84 (0.80-0.85)	5.47 (4.8-6.3)	0.13 (0.1-0.2)	0.73
27/28	0.92 (0.90-0.95)	0.78 (0.76-0.81)	4.29 (3.8-4.8)	0.10 (0.07-0.1)	0.70
28/29	0.96 (0.93-0.97)	0.75 (0.72-0.77)	3.81 (3.4-4.2)	0.06 (0.04-0.1)	0.71
29/30	0.97 (0.96-0.99)	0.61 (0.58-0.64)	3.01 (2.8-3.3)	0.05 (0.03-0.08)	0.58

+LR, -LR: positive and negative likelihood ratios; Sn: sensitivity; Sp: specificity.

**Figure 4:**
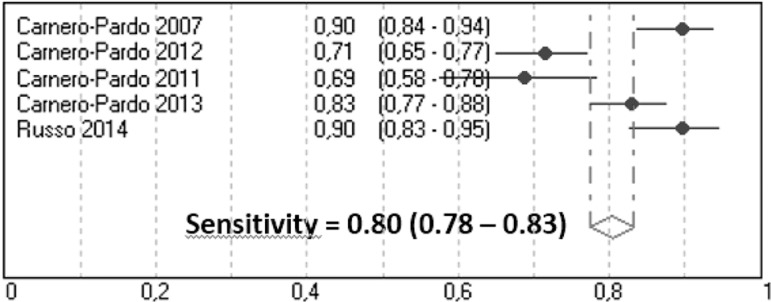
Meta-analysis of sensitivity for cognitive impairment.

**Figure 5:**
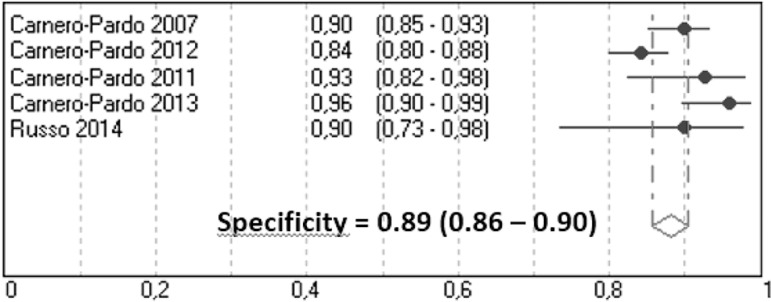
Meta-analysis of specificity for cognitive impairment.

**Cognitive impairment.** Sn and Sp diagnostic parameters for cognitive impairment are shown in Table 1. In all studies, subjects with dementia are included in cognitive impairment group. In the random-effects meta-analysis, Sn was 0.80 (95% CI, 0.77-0.92) with evidence of significant heterogeneity between studies (Q=34.8, p<0.001) (Figure 4), and Sp was 0.88 (95% CI, 0.86-0.90) also showing significant heterogeneity (Q=13.7, p<0.01) (Figure 5).

In the meta-analysis of individual data, a total of 1565 subjects were included (766 without cognitive impairment and 799 with cognitive impairment); the aROC was 0.91 (95% CI, 0.93-0.92) and the cut-off point maximizing the Sn + Sp value was 28/29, with estimated Sn and Sp of 0.79 (95% CI, 0.76-0.81) and 0.88 (95% CI, 0.86-0.90), respectively. Diagnostic accuracy parameters (Sn, Sp, +LR, -LR and Youden index) for different cut-offs of pooled individual data are shown in Table 3.

**Table 3:** Results of the meta-analysis of individual participant data based on parameters estimated for cognitive impairment

Cut-off	Sn (95 %CI )	Sp (95 %CI )	+LR (95 %CI )	-LR (95 %CI )	Youden index
26/27	0.68 (0.65-0.72)	0.95 (0.93-0.96)	12.8 (9.5-17.3)	0.33 (3-0.4)	0.63
27/28	0.75 (0.72-0.79)	0.91 (0.89-0.93)	8.7 (6.9-11.0)	0.28 (0.2-0.3)	0.66
28/29	0.79 (0.76-0.81)	0.88 (0.86-0.90)	6.7 (5.5-8.1)	0.24 (0.2-0.3)	0.67
29/30	0.83 (0.81-0.86)	0.82 (0.80-0.85)	4.73 (4.0-5.5)	0.20 (0.2-0.2)	0.65
30/31	0.88 (0.86-0.90)	0.77 (0.74-0.80)	3.83 (3.4-4.4)	0.16 (0.1-0.2)	0.65
31/32	0.91 (0.88-0.93)	0.68 (0.65-0.72)	2.86 (2.6-3.2)	0.14 (0.1-0.2)	0.59
32/33	0.93 (0.91-0.95)	0.58 (0.55-0.62)	2.25 (2.1-2.4)	0.12 (0.09-0.2)	0.51

+LR, -LR: positive and negative likelihood ratios; Sn: sensitivity; Sp: specificity.

The meta-analysis of individual data also allowed estimation of specific LRs for different cut-off points or intervals for dementia (Table 4) and cognitive impairment (Table 5).

**Table 4:** Post-test probabilities (predictive values) of the Phototest for different cut-off intervals for dementia.

Phototest	Dementia	Cognitive impairment	LR
0-20	220	16	32.93
21-23	108	51	5.07
24-26	81	112	1.73
27-29	37	176	0.50
≥30	15	769	0.05
**Total**	**461**	**1104**	

LR: likelihood ratios.

**Table 5:** Post-test probabilities (predictive values) of the Phototest for different cut-off intervals for cognitive impairment.

Phototest	Dementia	Cognitive impairment	LR
0-24	233	15	27.50
25-27	168	51	3.16
28-30	105	100	1.00
31-33	57	217	0.25
≥34	39	373	0.10
**Total**	**799**	**766**	

LR: likelihood ratios.

## DISCUSSION

In this systematic review, we found a total of 8 studies concerning Phototest diagnostic accuracy, 6 of which were included in the meta-analysis,^2,8,12,13,15,16^ whereas 2 studies were excluded because they employed a preliminary pilot version of the test.^1,7^ Results showed acceptable diagnostic accuracy parameters for dementia, with better Sp (0.87, 95% CI 0.82-0.88) than Sn (0.85, 95% CI 0.82-0.88). Estimates for cognitive impairment also showed higher Sp (0.88, 95% CI 0.85-0.91) than Sn (0.80, 95% CI 0.77-0.92). Similar results were found in the meta-analysis of individual participant data, with optimal cut-off points of 26/27 for dementia (Sn=0.89, 95% CI 0.85-0.91; Sp=0.84, 95% CI 0.82-0.86) and 28/29 for cognitive impairment (Sn=0.79, 95% CI 0.76-0.81; Sp=0.88, 95% CI 0.86-0.90). In the latter analysis, we calculated clinically relevant score intervals and their corresponding LRs. This allowed estimation of pooled post-test probability for known sample prevalence.^22^

Studies were heterogeneous regarding number of subjects included, variable settings, cut-off points used and quality of data. They were however, highly homogeneous with respect to diagnostic criteria since, except for the Argentine-2 study, authors used the same diagnostic criteria, namely: DSM-IV-TR^17^ for dementia and MCI criteria from the Spanish Neurological Society^18^ for cognitive impairment. The Argentine-2 study only included patients with probable Alzheimer´s disease and single domain amnestic MCI.

It is known that the Mini-Mental State Examination (MMSE)^23^ is the most commonly used cognitive screening test. However, a meta-analysis of the accuracy of the MMSE^24^ revealed its very limited value in detecting dementia (Sn=0.77, 95% CI 0.70-0.83; Sp=0.90, 95% CI 0.82-0.95), and particularly MCI (Sn=0.67, 95% CI 0.50-0.82; Sp=0.78, 95% CI 0.62-0.90). These results are in line with a side-by-side comparison of the two instruments, in which the Phototest proved to be more efficient due to superior effectiveness and lower cost than the MMSE.^13^ In addition to lower effectiveness, the MMSE has numerous other limitations (lack of standardization, application time, influence of socio-educational variables and copyright). These drawbacks have led one of the authors to suggest this could be the right time to reject this test.^25^

The Eurotest^26^ is another instrument which can be used in illiterate persons that is independent of socio-educational factors and has shown similar diagnostic accuracy to the Phototest in a recent meta-analyses^27^ (aROC=0.94 in both studies) exhibiting higher Sn (0.91[0.88-0.94]) and lower Sp (0.84[0.82-0.86]). Another study with a side-by-side comparison of the Eurotest and Phototest showed the same effectiveness.^14^ If we consider that the Eurotest requires double the time to apply (6 to 8 min) than the Phototest (<3 min), despite being equally effective, the latter proves to be more efficient and useful in clinical practice.

The Clock Drawing Test^28^ is another brief and widely-used cognitive test. However, in a recent systematic review,^29^ a quantitative meta-analysis was unfeasible due to the large variety of correction systems applied in publications. Two recent studies have shown modest utility both in specialized (aROC=0.88 for dementia, aROC=0.82 for cognitive impairment)^30^ and in primary care settings (aROC=0.84 for CI).^31^ These poor results may be linked to low educational level of the study populations, since it is known that education greatly influences test results.^29,32^

The main limitations of this meta-analysis are the study heterogeneity and the small sample size of some of the studies included. Its strengths include the homogeneity of the diagnostic criteria used, the varied study locations which comprised multiple clinical settings, and above all, the individual participant data analysis that included data from all studies, bar one. This meta-analysis allowed robust estimation of diagnostic accuracy parameters for either point estimate or interval results.

In conclusion, this meta-analysis showed that the Phototest offers adequate diagnostic accuracy for cognitive impairment, and particularly for dementia, that is similar or superior to other instruments widely used in our milieu. Additionally, it is simple, brief, uninfluenced by educational variables and can even be used in individuals who are illiterate. These advantages make it attractive for use in populations with low educational level and/or in time-limited settings such as primary care.
